# Commentary: Localized vs. Systematic Neurodegeneration: A Paradigm Shift in Understanding Neurodegenerative Diseases

**DOI:** 10.3389/fnsys.2017.00091

**Published:** 2017-12-07

**Authors:** Bianca Norrara, Jhulimar G. Doerl, Fausto P. Guzen, Jose Rodolfo Lopes P. Cavalcanti, Marco Aurelio M. Freire

**Affiliations:** Laboratory of Experimental Neurology, Department of Biomedical Sciences, Faculty of Healthy Sciences, University of the State of Rio Grande do Norte (UERN), Mossoró, Brazil

**Keywords:** neurodegeneration, nervous system, excitotoxicity, inflammation, therapeutics

Neurodegenerative disorders are one of the most important causes of disability in humans (Batista and Pereira, 2016), causing a limitation in the professional, social, and personal activities of its sufferers and also impacting their families, as well as a severe socioeconomic burden to the social security (Uryu et al., 2010; Kivimaki et al., 2015). For instance, only in United States of America (USA) the annual cost of medical care related to head injury reaches around U$200 million every year (see Humphreys et al., 2013 for a review), which varies depending on the severity of lesion, population evaluated and time period (Dismuke et al., 2015).

Neurodegeneration can be induced by acute injury or chronic diseases. The first condition is elicited by the brain and spinal cord trauma and involves two main events: (1) Primary neuronal degeneration, which induces an abrupt process of tissue degradation and cell death by a mechanical disturbance of the nervous tissue, causing breakdown of the blood-brain barrier (BBB), intense inflammatory response and tissue swelling, with an irremediable tissue damage (Maas et al., 2008); and (2) Secondary neuronal degeneration, a condition correlated to the intensity of the primary insult, which occurs gradually and involves impairment of nervous tissue initially spared by the primary lesion. Inflammation and excitotoxicity are factors involved with the spreading of the primary lesion, ultimately affecting the healthy tissue (Choi, 1992; Morganti-Kossmann et al., 2002; Guimaraes et al., 2009; Freire, 2012). Inflammatory response, a physiological event that aims to safeguarding the tissue against harmful agents, promoting healing, and tissue repair, can have detrimental effects when becomes exacerbated, generating imbalanced production of pro-inflammatory cytokines such as tumor necrosis factor alpha (TNF-α) and interleukin-1 beta (IL-1β) that further contribute to tissue impairment in both central and peripheral tissues (Allan and Rothwell, 2001; Araujo et al., 2014; Friese et al., 2014). Excitotoxicity, in turn, possesses a pivotal role in the impairment of the nervous system, increasing tissue lesion by an overactivation of glutamatergic receptors, with a consequent influx of ion calcium and production of free radicals, leading to apoptotic cell death (Manev et al., 1989; Dong et al., 2009).

One of the significant features of acute brain injury is the phenomenon called diffuse axonal injury, characterized by a widespread pattern of lesion that causes a disturbance in the axonal physiology, especially in the white matter (Johnson et al., 2013), normally presenting a fast progression, with well-evident motor and/or cognitive disorders (Lin and Wen, 2013). In addition, traumatic brain injury is pointed as an important risk factor for the development of neurodegenerative diseases (Gupta and Sen, 2016).

In chronic neurodegenerative diseases, such as Parkinson's disease and Amyotrophic Lateral Sclerosis, conversely, it is possible to determine accurately the initial site where neuronal death begins. In Parkinson's disease, for instance, dopaminergic neurons of the midbrain are primarily affected (Sulzer and Surmeier, 2013). Following the progression of the pathology, other neuronal groups also collapse (Sulzer and Surmeier, 2013). Likewise acute brain lesions, impairment of normal tissue physiology in chronic brain pathologies is also related to inflammatory response and excitotoxicity (Akiyama et al., 2000; Gao and Hong, 2008; Ambrosi et al., 2014; Amor et al., 2014; Santos et al., 2014).

As abovementioned, acute and chronic degenerations present particularities regarding both location and kinetics of tissue impairment. In this context, a recent opinion article published by Bayati and Berman (2017) in Frontiers in System Neuroscience raises an important point: mechanistic progression and the characteristic patterns of degeneration of neurological diseases are critical points to be approached in order to establish an effective diagnostic and/or proper treatment of their symptoms (Bayati and Berman, 2017). As pointed by authors, a comprehensive characterization of specific types of degenerative pathologies regarding their location and progression of cell loss would be valuable to researchers and medical professionals in order to provide an early diagnosis, aiming at least to slow down the progression of the diseases. For instance, Alzheimer's disease, the most prevalent chronic neurodegenerative disease, presents a pattern of global brain impairment, and because of this characteristic, is not possible to establish a treatment focused in a specific brain region, as made for Parkinson's disease. Thus, the localization of function, as stated by Bayati and Berman, is advantageous to an early characterization of cell loss and tissue impairment in specific regions of the brain in some neurodegenerative pathologies.

In conclusion, Bayati and Berman's paper offers a new and interesting perspective about how neurodegenerative diseases can be approached, in light of their systematic or localized characteristics. So, a proper understanding concerning both acute and chronic pathological conditions is a key factor for the development of effective progress in therapeutics.

## Author contributions

All authors listed have made a substantial, direct and intellectual contribution to the work, and approved it for publication.

### Conflict of interest statement

The authors declare that the research was conducted in the absence of any commercial or financial relationships that could be construed as a potential conflict of interest.

## References

[B1] Akiyama H. Barger S. Barnum S. Bradt B. Bauer J. Cole G. M.. (2000). Inflammation and Alzheimer's disease. Neurobiol. Aging 21, 383–421. 10858586 10.1016/s0197-4580(00)00124-x PMC3887148

[B2] Allan S. M. Rothwell N. J. (2001). Cytokines and acute neurodegeneration. Nat. Rev. Neurosci. 2, 734–744. 10.1038/35094583 11584311

[B3] Ambrosi G. Cerri S. Blandini F. (2014). A further update on the role of excitotoxicity in the pathogenesis of Parkinson's disease. J. Neural Transm. 121, 849–859. 10.1007/s00702-013-1149-z 24380931

[B4] Amor S. Peferoen L. A. Vogel D. Y. Breur M. van der Valk P. Baker D.. (2014). Inflammation in neurodegenerative diseases–an update. Immunology 142, 151–166. 10.1111/imm.12233 24329535 PMC4008224

[B5] Araujo R. F. Jr. Reinaldo M. P. O. Brito G. A. Cavalcanti P. F. Freire M. A. M. Medeiros C. A.. (2014). Olmesartan decreased levels of IL-1β and TNF-α, down-regulMMP-2, MMP-9, COX-2, RANK/RANKL and up-regulated SOCs-1 in an intestinal mucositis model. PLoS ONE 9:e114923. 10.1371/journal.pone.0114923 25531650 PMC4273993

[B6] Batista P. Pereira A. (2016). Quality of life of patients with neurodegenerative diseases. J. Neurol. Neurosci. 7, 1–7. 10.21767/2171-6625.100074

[B7] Bayati A. Berman T. (2017). Localized vs. systematic neurodegeneration: a paradigm shift in understanding neurodegenerative diseases. Front. Syst. Neurosci. 11:62. 10.3389/fnsys.2017.00062 28878634 PMC5572257

[B8] Choi D. W. (1992). Excitotoxic cell death. J. Neurobiol. 23, 1261–1276. 10.1002/neu.480230915 1361523

[B9] Dismuke C. E. Walker R. J. Egede L. E. (2015). Utilization and cost of health services in individuals with traumatic brain injury. Glob. J. Health Sci. 7, 156–169. 10.5539/gjhs.v7n6p156 26153156 PMC4803849

[B10] Dong X. X. Wang Y. Qin Z. H. (2009). Molecular mechanisms of excitotoxicity and their relevance to pathogenesis of neurodegenerative diseases. Acta Pharmacol. Sin. 30, 379–387. 10.1038/aps.2009.24 19343058 PMC4002277

[B11] Freire M. A. M. (2012). Pathophysiology of neurodegeneration following traumatic brain injury. West Indian Med. J. 61, 751–755. 10.7727/wimj.2012.003 23620976

[B12] Friese M. A. Schattling B. Fugger L. (2014). Mechanisms of neurodegeneration and axonal dysfunction in multiple sclerosis. Nat. Rev. Neurol. 10, 225–238. 10.1038/nrneurol.2014.37 24638138

[B13] Gao H. M. Hong J. S. (2008). Why neurodegenerative diseases are progressive: uncontrolled inflammation drives disease progression. Trends Immunol. 29, 357–365. 10.1016/j.it.2008.05.002 18599350 PMC4794280

[B14] Guimarães J. S. Freire M. A. M. Lima R. R. Souza-Rodrigues R. D. Costa A. M. Santos C. D.. (2009). Mechanisms of secondary degeneration in the central nervous system during acute neural disorders and white matter damage. Rev. Neurol. 48, 304–310. Available online at: https://www.neurologia.com/articulo/2008512/eng 19291655

[B15] Gupta R. Sen N. (2016). Traumatic brain injury: a risk factor for neurodegenerative diseases. Rev. Neurosci. 27, 93–100. 10.1515/revneuro-2015-0017 26352199

[B16] Humphreys I. Wood R. L. Phillips C. J. Macey S. (2013). The costs of traumatic brain injury: a literature review. Clinicoecon. Outcomes Res. 5, 281–287. 10.2147/CEOR.S44625 23836998 PMC3699059

[B17] Johnson V. E. Stewart W. Smith D. H. (2013). Axonal pathology in traumatic brain injury. Exp. Neurol. 246, 35–43. 10.1016/j.expneurol.2012.01.013 22285252 PMC3979341

[B18] Kivimäki M. Vineis P. Brunner E. J. (2015). How can we reduce the global burden of disease? Lancet 386, 2235–2237. 10.1016/S0140-6736(15)00129-4 26364543

[B19] Lin Y. Wen L. (2013). Inflammatory response following diffuse axonal injury. Int. J. Med. Sci. 10, 515–521. 10.7150/ijms.5423 23532682 PMC3607236

[B20] Maas A. I. Stocchetti N. Bullock R. (2008). Moderate and severe traumatic brain injury in adults. Lancet Neurol. 7, 728–741. 10.1016/S1474-4422(08)70164-9 18635021

[B21] Manev H. Favaron M. Guidotti A. Costa E. (1989). Delayed increase of Ca^2+^ influx elicited by glutamate: role in neuronal death. Mol. Pharmacol. 36, 106–112. 2568579

[B22] Morganti-Kossmann M. C. Rancan M. Stahel P. F. Kossmann T. (2002). Inflammatory response in acute traumatic brain injury: a double-edged sword. Curr. Opin. Crit. Care 8, 101–105. Available online at: https://insights.ovid.com/pubmed?pmid=12386508 12386508 10.1097/00075198-200204000-00002

[B23] Santos J. R. Gois A. M. Mendonca D. M. Freire M. A. M. (2014). Nutritional status, oxidative stress and dementia: the role of selenium in Alzheimer's disease. Front. Aging Neurosci. 6:206. 10.3389/fnagi.2014.00206 25221506 PMC4147716

[B24] Sulzer D. Surmeier D. J. (2013). Neuronal vulnerability, pathogenesis, and Parkinson's disease. Mov. Disord. 28, 715–724. 10.1002/mds.25095 23589357

[B25] Uryu K. Haddix T. Robinson J. Nakashima-Yasuda H. Lee V. M. Trojanowski J. Q. (2010). Burden of neurodegenerative diseases in a cohort of medical examiner subjects. J. Forensic Sci. 55, 642–645. 10.1111/j.1556-4029.2010.01347.x 20345790 PMC2951130

